# Carvacrol and Thymol Enhance the Quality of Beni Arouss Buck Semen Stored at 4 °C Thanks to Their Antimicrobial Properties

**DOI:** 10.3390/vetsci11090406

**Published:** 2024-09-03

**Authors:** Amr Kchikich, Nathalie Kirschvink, Marianne Raes, Samira El Otmani, Youssef Chebli, Jean-Loup Bister, Bouchra El Amiri, Said Barrijal, Mouad Chentouf

**Affiliations:** 1 Department of Biology, Abdelmalek Essaadi University, Tangier 93000, Morocco; kchikch.amr@gmail.com (A.K.); s.barrijal@uae.ac.ma (S.B.); 2 Regional Center of Agricultural Research of Tangier, National Institute of Agricultural Research, Rabat 10090, Morocco; samira.elotmani@inra.ma (S.E.O.); youssef.chebli@inra.ma (Y.C.); 3 Department of Medicine, Namur Research Institute for Life Sciences (NARILIS), University of Namur, 5000 Namur, Belgium; nathalie.kirschvink@unamur.be; 4 Department of Veterinary Medicine, Namur Research Institute for Life Sciences (NARILIS), University of Namur, 5000 Namur, Belgium; marianne.raes@unamur.be (M.R.); jean-loup.bister@unamur.be (J.-L.B.); 5 Regional Center of Agricultural Research Settat, National Institute of Agricultural Research, Rabat 10090, Morocco; bouchra.elamiri@inra.ma

**Keywords:** Beni Arous bucks, carvacrol, thymol, semen conservation

## Abstract

**Simple Summary:**

This study investigates how carvacrol and thymol affect the quality of semen from Beni Arouss bucks stored in skim milk at 4 °C. Semen from eight bucks was collected weekly for 11 weeks, pooled, and divided into three groups: one diluted in skim milk, and the others diluted in skim milk supplemented with 200 µM of carvacrol and thymol. Sperm motility, viability, abnormalities, membrane integrity, lipid damage, and bacterial growth were assessed during 48 h of storage at 4 °C. After 48 h, carvacrol improved sperm motility, viability, and reduced bacterial growth and lipid damage. Thymol showed similar benefits but did not enhance progressive motility. These beneficial effects are due to the antimicrobial properties of these two compounds, offering potential benefits for livestock breeding.

**Abstract:**

This study aims to investigate the impact of carvacrol and thymol on the quality of Beni Arouss buck semen stored in skim milk at 4 °C. Ejaculates were collected from eight Beni Arouss bucks weekly for 11 weeks, pooled, and then divided into three equal parts. Samples were diluted to 400 × 10^6^ sperm/mL in skim milk (control) and skim milk supplemented with a single dose of 200 µM carvacrol and thymol each. Evaluations of sperm motility, viability, abnormalities, membrane integrity, lipid peroxidation, and bacterial growth were conducted at 0, 6, 24, and 48 h of liquid storage at 4 °C. After 48 h of storage, the results indicate that the addition of carvacrol positively influences total and progressive motility and viability. However, it also leads to a decrease in lipid peroxidation and bacterial growth compared to the control group (*p* < 0.05). Thymol showed similar results to carvacrol, except for progressive motility (*p* > 0.05). Bacterial growth was negatively correlated with total and progressive motility and viability (*p* < 0.05), while no correlation between lipid peroxidation and these parameters was observed (*p* > 0.05). In conclusion, the addition of carvacrol and thymol to skim milk extender moderately improves the quality of Beni Arouss buck semen after 48 h storage at 4 °C due to its antimicrobial activity.

## 1. Introduction

Artificial insemination is a widely used technique for spreading genetic progress and improving herd productivity. However, in goats, its use is relatively limited and does not offer the same level of fertility as in cattle [1]. The successful use of this technique depends mainly on the quality of the stored semen [2]. It is therefore essential to optimize sperm storage protocols by extending sperm life without compromising its functionality. Deterioration of sperm quality is inevitable during storage, with bacterial proliferation and oxidative stress being the main causes of this degradation [3].

Bacterial proliferation is an important factor affecting sperm quality, because semen is routinely collected and processed under non-sterile conditions. Moreover, the preservation of diluted semen at moderate temperatures, with nutrient-rich extenders, creates favorable conditions for considerable bacterial multiplication [4]. Bacterial proliferation leads to sperm agglutination, decreased sperm motility, viability, membrane integrity and acrosomal integrity acrosome damage [5].

Oxidative stress is another important factor affecting semen quality. Sperm has defense mechanisms against free radicals, including endogenous antioxidants [6]. However, despite these protective systems, sperm cells remain highly sensitive to free radicals. Recent research has highlighted the role of oxidative stress, resulting from an imbalance between antioxidants and reactive oxygen species (ROS), on the deterioration of sperm quality [7]. All cellular constituents, such as proteins, lipids, and nucleic acids, are vulnerable to oxidative stress caused by ROS, with serious consequences for sperm membrane integrity and motility [8]. This ultimately accelerates the process of sperm degradation, reducing its viability and ability to fertilize [9].

In recent years, natural compounds extracted from plants have attracted great interest in semen preservation due to their varied biological activities, notably their antibacterial and antioxidant mechanisms [10,11]. Essential oils have specific properties linked to their main constituents. For example, carvacrol content in *Thymus vulgaris* (46%) contributes to its many properties [12], while *Lippia thymoides* essential oil owes its effects mainly to its thymol content (62%) [13]. Although many compounds are present, one or two often dominate the oil’s physiological action. This chemical complexity helps explain the marked effects of essential oils, even when certain components are present in low concentrations, such as bergapten in bergamot oil (around 0.3%) [14].

This paper focuses on Beni Arouss goats, an autochthonous breed of Northern Morocco officially recognized by the Moroccan Ministry of Agriculture. The Beni Arouss breed is a mixed-purpose goat known for its red coat and good build and reared under an extensive production system in the Rif mountains. Milk yield is estimated at 55 kg per lactation and used for suckling kids and making traditional cheese [15]. This breed is under a specific breeding program carried out at the farm level by the Sheep and Goat National Association. Improving Beni Arouss buck semen conservation is essential to support this program by improving the success rate of artificial insemination. A previous study showed that the addition of 0.01% of *Thymus satureioides* essential oil to skim milk extender effectively preserves buck semen for 48 h at 4 °C; this essential oil contains mainly carvacrol and thymol, representing 31% and 28% respectively [16]. In the present study, we selected a concentration of 200 µM for carvacrol and thymol to reflect the proportions found in *Thymus satureioides* essential oil at 0.01%, aiming to assess their impact on Beni Arous buck semen quality during 48 h storage at 4 °C. The use of these specific compounds allows for the avoiding of any variability in essential oil composition due to the variety, crop management, or climatic and edaphic conditions.

## 2. Materials and Methods

### 2.1. Antioxidant Activity Assessment

The free radical scavenging activity of skim milk, serving as a control, and skim milk supplemented with carvacrol (200 µM, Sigma-Aldrich, 42632, Taufkirchen, Germany) and thymol (200 µM, Sigma-Aldrich, 72477, Taufkirchen, Germany) was assessed using 2,2-diphenyl-1-picrylhydrazyl (DPPH) and replicated five times [17].

### 2.2. Semen Collection and Evaluation

**Figure 1:**
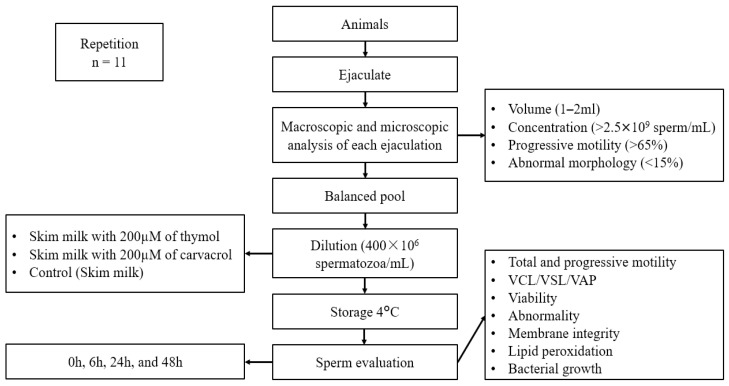
Experimental design.

The study was carried out at Boukhalef experimental station belonging to the National Institute of Agricultural Research (35°43′48″ N Latitude, 5°52′58″ W Longitude) under a natural photoperiod from March to May. Ejaculates from eight Beni Arouss bucks, aged between 30 and 54 months, a North Moroccan autochthonous goat breed [18], were collected weekly on the same day over 11 weeks using an artificial vagina. Among the 88 ejaculates collected, 80 met the following criteria and were used in this experiment: a volume of 1 to 2 mL, a concentration greater than 2.5 × 10^9^ sperm/mL, progressive motility exceeding 65%, and more than 85% normal morphology. The ejaculate volume was determined using a conical tube graduated at 0.1 mL, sperm concentration was measured by spectrophotometry (IMV Technologies, L’Aigle, France), and progressive motility was evaluated using a computer-assisted sperm analysis (CASA) system (ISAS^®^, Proiser R + D SL, Leganes, Spain). Sperm abnormalities were identified via eosin–nigrosin staining (Minitube^®^, Tiefenbach, Germany). Equal volumes of each ejaculate were then placed in a water bath at 37 °C, combined to minimize individual variability, and divided into three equal parts (carvacrol, thymol, and control), diluted to a concentration of 400 × 10^6^ sperm/mL, and stored at 4 °C. Semen quality was assessed after being stored at 4 °C for 0, 6, 24, and 48 h (Figure 1).

### 2.3. Sperm Motility Evaluation

Sperm motility was evaluated using a computer-assisted sperm-analysis system provided by ISAS (Proiser R + D SL, Leganes, Spain). This setup includes a phase-contrast optical microscope (UB203i, Proiser R + D SL, Leganes, Spain) connected to a digital camera and a computer. Before the analysis, the sperm concentration was adjusted to 30 × 10^6^ sperm/mL using a diluent. Images were taken from 3 μL sperm aliquots placed on a 20 μm deep-chamber slide (SpermTrack 20, Proiser R + D SL, Leganes, Spain) on a stage heated to 37 °C. To assess total and progressive motility (%), curvilinear velocity (VCL, µm/s), straight-line velocity (VSL, µm/s), and average path velocity (VAP, µm/s), a negative phase-contrast microscope with 10× magnification was used, analyzing approximately 200 spermatozoa across six different fields in each sample [19].

### 2.4. Viability and Abnormality

The viability and abnormality of spermatozoa (%) were evaluated using the eosin–nigrosin staining method (Minitube^®^, Germany), following the procedure outlined by Evans and Maxwell [20]. A 3 µL sample (containing 400 × 10^6^ sperm/mL) was mixed with 2% eosin and 4% nigrosin (*v*/*v*) on a slide warmed to 37 °C to prepare sperm suspension smears. The stain was spread immediately using another slide. A total of 400 spermatozoa were examined across multiple microscopic fields at 600× magnification to calculate the percentages of live, dead, normal, and abnormal spermatozoa. Sperm with the head stained partially or entirely purple were considered dead, whereas only the unstained sperm were regarded as alive. Normal spermatozoa have an oval head and a long tail. The morphological categories used in this study were as follows: 1. abnormal heads (including amorphous heads, pyriform heads, elongated heads, round heads, acrosome defects, vacuolated heads), 2. abnormal midpieces, (including thick midpiece, thin midpiece bent neck, excessive residual cytoplasm, asymmetrical neck insertion) 3. abnormal tail (including double tail, coiled tail, short tail) [21].

### 2.5. Membrane Integrity

The sperm membrane integrity was assessed using the hypo-osmotic swelling test (HOST). In accordance with Revell and Mrode’s protocol [22], 300 µL of 100 mOsm HOST solution was incubated with 30 µL of sperm (400 × 10^6^ spermatozoa/mL) for 60 min at 37 °C. Using the procedure described by Buckett et al. [23], 200 spermatozoa were counted with coiled tails under a microscope at 600× magnification.

### 2.6. Lipid Peroxidation

Using the thiobarbituric acid reaction substances (TBARS) technique, malondialdehyde (MDA) content was quantified as an indication of lipid peroxidation [24]. In short, 500 µL of the diluted sample was mixed with 1 mL of the thiobarbituric acid combination. The mixture was heated to 100 °C for 10 min, followed by cooling in an ice bath to stop the reaction. Centrifugation was then performed to remove precipitates. TBARS levels were quantified using a spectrophotometer (Jenway 6310, Keison Products, Chelmsford, UK), with results expressed as nM TBARS per 10^8^ spermatozoa. Calibration was performed using an external standard derived from MDA produced by the hydrolysis of 1,1,3,3-tetraethoxypropane.

### 2.7. Bacterial Growth Assessment

Agar (Mueller–Hinton, Biokar diagnostics) media was used to assess total aerobic microbial counts in semen samples during storage. In brief, 1 mL of each semen sample was mixed with sterile PBS (1:10 dilution), and homogenized using a vortex. Subsequently, diluted semen (100 μL) was inoculated onto Mueller–Hinton agar plates in triplicate, followed by aerobic incubation at 37 °C. Colony-forming units (CFU) were enumerated after 48 h of incubation [25].

### 2.8. Statistical Analysis

Percentage data were transformed using arcsine normalization (root (p)) to stabilize variance, normalize distributions, and enhance interpretability. Following this transformation, the datasets were analyzed using ANOVA within a general linear model framework in SAS, considering the fixed effects of treatment, storage duration, and their interaction. Differences between means were assessed using the Tukey test. Pearson correlation coefficients were calculated for all variables. Data analysis was carried out using SAS 9.4 software, with statistical significance set at a *p*-value of less than 0.05.

## 3. Results

### 3.1. Antioxidant Activity

Free radical-scavenging activity by DPPH showed different results between sperm-free extenders, with values of 49.58%, 56.44% and 54.35% in control skim milk and skim milk supplemented with carvacrol and thymol, respectively.

### 3.2. Sperm Motility

Results for total and progressive motility showed significant variations according to treatment and storage duration (Table 1).

All treatments showed a decrease in total and progressive motility over 48 h of storage. Semen treated with carvacrol and thymol showed a less pronounced decline compared to the control group in total motility (*p* < 0.05). Additionally, the carvacrol-treated group showed higher levels of progressive motility than the control and thymol group after 48 h of storage (*p* < 0.05).

Kinetic parameters showed significant variations depending on the treatments applied and storage duration (Table 2).

There were no significant differences in kinetic parameters between treatments at 0 h; variations appeared after 6 hours’ storage, with carvacrol affecting all kinetic parameters and thymol influencing VCL and VAP (*p* < 0.05). After 48 hours’ storage, carvacrol and thymol showed higher values than the control group for VCL, VSL, and VAP (*p* < 0.05).

### 3.3. Viability and Abnormalities

Viability decreased for all treatments (Control, carvacrol, and thymol) at all storage durations (*p* < 0.05; Table 3).

Treatments with carvacrol and thymol improved viability compared to the control at 48 h (*p* < 0.05). The percentage of abnormal spermatozoa revealed a significant increase over time for all treatments at all storage times (*p* < 0.05; Table 3). No significant variation between treatments was observed over the entire sperm storage period (*p* > 0.05).

### 3.4. Membrane Integrity and Lipid Peroxidation

The membrane integrity showed a significant decrease during storage for all treatments (carvacrol, thymol, and control) (*p* < 0.05). However, no significant differences were detected between treatments (Table 4).

The MDA concentration increased significantly during storage for all treatments (Table 4). The carvacrol-enriched treatment showed the lowest MDA levels, followed by thymol and control at 24 h and 48 h storage at 4 °C (*p* < 0.05).

### 3.5. Bacterial Growth

Bacterial growth showed a significant increase in CFU/mL during storage for all treatments. Moreover, it should be noted that bacterial growth was generally less pronounced with the carvacrol and thymol treatments than with the control group (*p* < 0.05). Remarkably, the antimicrobial efficacy of carvacrol was superior to that of thymol at 6 h, 24 h, and 48 h storage (*p* < 0.05, Table 5).

### 3.6. Correlation between Sperm Quality Parameters

Correlations between the different sperm quality parameters are shown in Table 6.

The results reveal significant positive correlations between total motility, progressive motility, kinematic parameters, viability, and sperm membrane integrity (*p* < 0.001). Abnormality showed a negative correlation with total motility, progressive motility, kinematic parameters, viability, and membrane integrity (*p* < 0.001), while positive correlations were found with lipid peroxidation and bacterial growth (*p* < 0.001). Furthermore, bacterial growth was negatively correlated with total motility, progressive motility, kinematic parameters, viability, and membrane integrity (*p* < 0.001).

## 4. Discussion

To the best of the authors’ knowledge, this is the first study analyzing the effect of thymol and carvacrol on goat semen conservation. Previous studies were conducted on boar [26,27], stallions [28] and humans [29], while others have investigated the effect of orally administered thymol and carvacrol on rat semen [30,31]. The results obtained in these studies showed varied trends. For example, the highest motility of boar spermatozoa after 24 h of storage was 25% with thymol and 40% with carvacrol compared to other substances such as ethylgallate, hydroquinone, and cnicin, but with no improvement compared to the control [26]. Furthermore, the addition of carvacrol did not influence porcine sperm motility, but at low concentrations it decreased ROS production, while 30 μM of carvacrol reduced membrane integrity and 25 μM decreased mitochondrial membrane potential [27]. Regarding thymol, (50 μM, 100 μM, and 150 μM) maintained total and progressive motility and kinematic parameters in stallion semen during all storage times (0, 24, and 48 h) without affecting fungal growth after 48 h of storage compared with the control [28]. Conversely, human spermatozoa’s percentage motility and viability decreased from a 200 μg/mL thymol concentration in a dose-dependent way [29].

Our results showed that the addition of carvacrol and thymol to skim milk improved its antioxidant activity and the highest value was observed by adding carvacrol with 56.44%. The antioxidant and antimicrobial properties of thymol and carvacrol have already been reported [32]. Al-Mansori et al. [33], examining the antioxidant activities of thymol and carvacrol, suggested that carvacrol had the highest DPPH radical scavenging activity. David [34] reported that carvacrol demonstrated strong antimicrobial activity against *Acinetobacter calcoacetica*, *Aeromonas hydrophila*, *Bacillus subtilis*, *Clostridium sporogenes*, and *Pseudomonas aeruginosa*. In contrast, the same author stated that thymol was more effective against *Citrobacter freundii*, *Enterobacter aerogenes*, *Klebsiella pneumoniae*, and *Escherichia coli*.

As expected, our findings showed that during storage there was a decline in total and progressive motility, kinematic parameters, viability, and membrane integrity, coupled with an increase in abnormalities, lipid peroxidation, and bacterial growth for all treatments (*p* < 0.05). After 48 h of storage, total and progressive motility, kinematic parameters, viability, and lipid peroxidation were better preserved by carvacrol and thymol. These results are in accordance with our previous findings [16] using 0.01% of *Thymus satureioides* essential oil (carvacrol 31%, thymol 28%). In contrast, and unlike *Thymus satureioides* essential oil, no significant effects on membrane integrity or sperm morphology were observed between treatments, despite a lower MDA concentration in the carvacrol and thymol group compared to the control. This result is most likely due to the limited antioxidant activity of carvacrol and thymol at the tested level protecting the sperm membrane and morphology against oxidation during storage. It appears that *Thymus satureioides* essential oil exhibits a higher antioxidant activity, likely as result of a synergistic interaction between carvacrol and thymol and other minor compounds [35].

The correlation results highlighted that carvacrol and thymol’s antimicrobial activity was responsible for the beneficial effect on sperm quality during storage, rather than their antioxidant properties. Indeed, lipid peroxidation, an indicative marker of oxidative stress, had no significant correlation with motility and viability, while bacterial growth showed a significant negative correlation with these parameters. This suggests that the high bacterial presence played a role in causing damage to sperm. The bacteriostatic effect of carvacrol and thymol at reduced concentrations succeeded in minimizing bacterial proliferation, thus preserving motility and viability compared with the control group after 48 h of storage. This result agrees with the findings of Mazurova et al. [36], who reported that carvacrol and thymol are the most effective natural substances for decontaminating boar semen against Gram-negative and Gram-positive bacteria. The reduced CFU concentration in the carvacrol group was probably responsible for a higher progressive motility compared to the thymol group. During the first 24 h, no significant change was noted between the different treatments for all parameters except for lipid peroxidation. This observation could be attributed to the low bacterial load compared to 48 h of storage, exceeding 20,000 CFU/mL. Previous studies highlighted that when the bacterial load exceeds this threshold, a deleterious impact on viability and motility occurs [37,38].

For successful fertilization, motility and viability remain the most crucial and visibly impacted parameters [39]. Our results showed that over the storage period, bacterial concentration increased while total motility, progressive motility, and viability decreased. It is pertinent to point out that in vitro studies have revealed that the mechanism of action of bacteria on sperm motility may result from attachment to sperm acrosome or flagellum receptors [40], secretion of toxic factors such as lipopolysaccharide endotoxin [41], or mitochondrial membrane damage and acrosome membrane rupture [42]. Reduced sperm viability due to bacterial contamination can occur through at least two mechanisms: (1) certain bacteria produce soluble spermatotoxic agents, such as sperm immobilization factors, which may lower viability by inhibiting mitochondrial ATPase activity [43], and (2) inflammation caused by bacteria can result in excessive reactive oxygen species (ROS) production, leading to sperm DNA damage and apoptosis [44].

Our study showed that the abnormality rate increased, and membrane integrity decreased over the storage time, and both parameters were positively correlated with bacterial growth and lipid peroxidation. Meanwhile, previous studies have highlighted the detrimental effects of various bacterial strains, including *Escherichia coli*, *Ureaplasma urealyticum*, *Chlamydia trachomatis*, *Mycoplasma hominis*, and *Neisseria gonorrhoeae*, on sperm morphology and membrane integrity [45]. Oxidative stress resulting from the overproduction of ROS can have detrimental consequences on the sperm membrane and its acrosomal region, leading to potential sperm morphological abnormalities [46,47].

## 5. Conclusions

The addition of carvacrol and thymol at 200 µM each to skim milk has beneficial effects on the preservation of Beni Arouss buck semen at 4 °C. This positive effect is due to the antibacterial activity of these two natural phenolic compounds. These results will support the artificial insemination and breeding programs of the Beni Arouss goat, an autochthonous breed in the North of Morocco.

## Figures and Tables

**Table 1:** Carvacrol, thymol, and 4 °C storage duration: their effects on Beni Arouss buck semen’s total and progressive motility (%).

Parameters	Treatments	Storage Duration				Interaction Storage Duration and Treatments
		0 h	6 h	24 h	48 h	
Total motility	Control	95 ± 1 ^aA^	91 ± 2 ^bA^	85 ± 2 ^cA^	72 ± 2 ^dB^	*p* < 0.0013
	Thymol	94 ± 2 ^aA^	92 ± 3 ^aA^	84 ± 3 ^bA^	77 ± 3 ^cA^	
	Carvacrol	94 ± 2 ^aA^	91 ± 2 ^bA^	84 ± 3 ^cA^	77 ± 3 ^dA^	
Progressive motility	Control	73 ± 3 ^aA^	67 ± 3 ^bA^	51 ± 3 ^cA^	45 ± 2 ^dB^	*p* < 0.1906
	Thymol	74 ± 3 ^aA^	67 ± 2 ^bA^	54 ± 4 ^cA^	48 ± 2 ^dAB^	
	Carvacrol	74 ± 4 ^aA^	69 ± 2 ^bA^	54 ± 4 ^cA^	49 ± 2 ^dA^	

The data are presented as Mean ± SD (*n* = 11). Different capital letters (^A,B^) signify a significant effect of treatments within the same storage duration (*p* < 0.05). Different lowercase letters (^a,b,c,d^) indicate a significant effect of storage duration for the same treatment (*p* < 0.05).

**Table 2:** Carvacrol, thymol, and 4 °C storage duration: their effects on Beni Arouss buck semen’s curvilinear velocity (VCL, µm/s), straight-line velocity (VSL, µm/s), and average pathway velocity (VAP, µm/s).

Parameters	Treatments	Storage Duration				Interaction Storage Duration and Treatments
		0 h	6 h	24 h	48 h	
VCL	Control	121 ± 6 ^aA^	116 ± 6 ^aB^	109 ± 7 ^bB^	100 ± 5 ^cB^	*p* < 0.1767
	Thymol	123 ± 6 ^aA^	121 ± 5 ^aA^	119 ± 12 ^aA^	110 ± 8 ^bA^	
	Carvacrol	128 ± 11 ^aA^	126 ± 4 ^abA^	120 ± 3 ^bcA^	114 ± 7 ^cA^	
VSL	Control	88 ± 9 ^aA^	83 ± 9 ^abB^	81 ± 3 ^bB^	74 ± 6 ^cC^	*p* < 0.4311
	Thymol	90 ± 5 ^aA^	85 ± 5 ^bAB^	84 ± 8 ^bAB^	78 ± 3 ^cB^	
	Carvacrol	93 ± 11 ^aA^	90 ± 4 ^abA^	87 ± 3 ^bcA^	83 ± 3 ^cA^	
VAP	Control	109 ± 7 ^aA^	102 ± 5 ^bB^	100 ± 6 ^bB^	88 ± 6 ^cB^	*p* < 0.1806
	Thymol	110 ± 7 ^aA^	107 ± 5 ^aA^	105 ± 11 ^aAB^	96 ± 9 ^bA^	
	Carvacrol	113 ± 9 ^aA^	111 ± 4 ^aA^	108 ± 3 ^aA^	98 ± 8 ^bA^	

The data are presented as Mean ± SD (*n* = 11). Different capital letters (^A,B,C^) signify a significant effect of treatments within the same storage duration (*p* < 0.05). Different lowercase letters (^a,b,c^) indicate a significant effect of storage duration for the same treatment (*p* < 0.05).

**Table 3:** Carvacrol, thymol, and 4 °C storage duration: their effects on Beni Arouss buck semen’s viability and abnormality (%).

Parameters	Treatments	Storage Duration				Interaction Storage Duration and Treatments
		0 h	6 h	24 h	48 h	
Viability	Control	96 ± 1 ^aA^	94 ± 2 ^bA^	89 ± 4 ^cA^	76 ± 3 ^dB^	*p* < 0.0091
	Thymol	98 ± 2 ^aA^	95 ± 2 ^bA^	87 ± 3 ^cA^	79 ± 3 ^dA^	
	Carvacrol	97 ± 2 ^aA^	94 ± 3 ^bA^	88 ± 3 ^cA^	81 ± 4 ^dA^	
Abnormality	Control	7 ± 2 ^cA^	8 ± 2 ^cA^	17 ± 1 ^bA^	23 ± 4 ^aA^	*p* < 0.4130
	Thymol	7 ± 3 ^cA^	9 ± 2 ^cA^	16 ± 3 ^bA^	20 ± 5 ^aA^	
	Carvacrol	7 ± 3 ^cA^	9 ± 3 ^cA^	15 ± 3 ^bA^	21 ± 4 ^aA^	

The data are presented as Mean ± SD (*n* = 11). Different capital letters (^A,B^) signify a significant effect of treatments within the same storage duration (*p* < 0.05). Different lowercase letters (^a,b,c,d^) indicate a significant effect of storage duration for the same treatment (*p* < 0.05).

**Table 4:** Carvacrol, thymol, and 4 °C storage duration: their effects on Beni Arouss buck semen’s membrane integrity (%) and malondialdehyde concentration (nM TBARS/10^8^ spermatozoa).

Parameters	Treatments	Storage Duration				Interaction Storage Duration and Treatments
		0 h	6 h	24 h	48 h	
Membrane integrity	Control	92 ± 2 ^aA^	84 ± 3 ^bA^	74 ± 3 ^cA^	65 ± 2 ^dA^	*p* < 0.5888
	Thymol	92 ± 1 ^aA^	86 ± 4 ^bA^	74 ± 3 ^cA^	65 ± 2 ^dA^	
	Carvacrol	92 ± 2 ^aA^	86 ± 3 ^bA^	75 ± 3 ^cA^	67 ± 2 ^dA^	
Lipid peroxidation	Control	0.4 ± 0.1 ^dA^	0.6 ± 0.1 ^cA^	1.1 ± 0.1 ^bA^	1.8 ± 0.1 ^aA^	*p* < 0.0001
	Thymol	0.4 ± 0.1 ^dA^	0.6 ± 0.1 ^cA^	0.8 ± 0.1 ^bB^	1.4 ± 0.1 ^aB^	
	Carvacrol	0.4 ± 0.1 ^dA^	0.5 ± 0.1 ^cA^	0.7 ± 0.1 ^bC^	1.2 ± 0.1 ^aC^	

The data are presented as Mean ± SD (*n* = 11). Different capital letters (^A,B,C^) signify a significant effect of treatments within the same storage duration (*p* < 0.05). Different lowercase letters (^a,b,c,d^) indicate a significant effect of storage duration for the same treatment (*p* < 0.05).

**Table 5:** Carvacrol, thymol, and 4 °C storage duration: their effects on Beni Arouss buck semen’s bacterial growth (CFU/mL).

Parameters	Treatments	Storage Duration				Interaction Storage Duration and Treatments
		0 h	6 h	24 h	48 h	
Bacterial growth	Control	930 ± 243 ^dA^	2762 ± 203 ^cA^	14,313 ± 861 ^bA^	24,097 ± 688 ^aA^	*p* < 0.0001
	Thymol	394 ± 66 ^dB^	1550 ± 291 ^cB^	9744 ± 892 ^bB^	22,930 ± 1003 ^aB^	
	Carvacrol	320 ± 123 ^dB^	1013 ± 110 ^cC^	7883 ± 492 ^bC^	20,086 ± 1241 ^aC^	

The data are presented as Mean ± SD (*n* = 11). Different capital letters (^A,B,C^) signify a significant effect of treatments within the same storage duration (*p* < 0.05). Different lowercase letters (^a,b,c,d^) indicate a significant effect of storage duration for the same treatment (*p* < 0.05).

**Table 6:** Correlation coefficients between Beni Arouss buck semen quality parameters stored at 4 °C.

	Total Motility	Progressive Motility	VCL	VSL	VAP	Viability	Abnormality	Membrane Integrity	Lipid Peroxidation	Bacterial Growth
Total motility	1	0.88 ***	0.65 ***	0.61 ***	0.72 ***	0.98 ***	−0.53 ***	0.76 ***	NS	−0.84 ***
Progressive motility		1	0.63 ***	0.61 ***	0.67 ***	0.90 ***	−0.51 ***	0.71 ***	NS	−0.78 ***
VCL			1	0.79 ***	0.82 ***	0.67 ***	−0.32 ***	0.49 ***	NS	−0.59 ***
VSL				1	0.78 ***	0.63 ***	−0.32 ***	0.50 ***	NS	−0.49 ***
VAP					1	0.73 ***	−0.37 ***	0.57 ***	NS	−0.60 ***
Viability						1	−0.51 ***	0.76 ***	NS	−0.82 ***
Abnormality							1	−0.89 ***	0.58 ***	0.44 ***
Membrane integrity								1	−0.42 ***	−0.61 ***
Lipid peroxidation									1	NS
Bacterial growth										1

NS indicates non-significant correlation, and *** *p* < 0.001 was the threshold for significant correlation coefficients. Abbreviations: VCL, curvilinear velocity (µm/s); VSL, straight-line velocity (µm/s); VAP, average pathway velocity (µm/s).

## Data Availability

Data are contained within the manuscript.
